# A two-branch trade-off neural network for balanced scoring sleep stages on multiple cohorts

**DOI:** 10.3389/fnins.2023.1176551

**Published:** 2023-06-23

**Authors:** Di Zhang, Jinbo Sun, Yichong She, Yapeng Cui, Xiao Zeng, Liming Lu, Chunzhi Tang, Nenggui Xu, Badong Chen, Wei Qin

**Affiliations:** ^1^Engineering Research Center of Molecular and Neuro Imaging of the Ministry of Education, School of Life Science and Technology, Xidian University, Xi’an, China; ^2^Intelligent Non-invasive Neuromodulation Technology and Transformation Joint Laboratory, Xidian University, Xi'an, China; ^3^South China Research Center for Acupuncture and Moxibustion, Medical College of Acu-Moxi and Rehabilitation, Guangzhou University of Chinese Medicine, Guangzhou, China; ^4^College of Artificial Intelligence, Xian Jiaotong University, Xian, Shaanxi, China

**Keywords:** polysomnography, sleep staging, deep learning, multiple cohorts, N1 sleep

## Abstract

**Introduction:**

Automatic sleep staging is a classification process with severe class imbalance and suffers from instability of scoring stage N1. Decreased accuracy in classifying stage N1 significantly impacts the staging of individuals with sleep disorders. We aim to achieve automatic sleep staging with expert-level performance in both N1 stage and overall scoring.

**Methods:**

A neural network model combines an attention-based convolutional neural network and a classifier with two branches is developed. A transitive training strategy is employed to balance universal feature learning and contextual referencing. Parameter optimization and benchmark comparisons are conducted using a large-scale dataset, followed by evaluation on seven datasets in five cohorts.

**Results:**

The proposed model achieves an accuracy of 88.16%, Cohen’s kappa of 0.836, and MF1 score of 0.818 on the SHHS1 test set, also with comparable performance to human scorers in scoring stage N1. Incorporating multiple cohort data improves its performance. Notably, the model maintains high performance when applied to unseen datasets and patients with neurological or psychiatric disorders.

**Discussion:**

The proposed algorithm demonstrates strong performance and generalizablility, and its direct transferability is noteworthy among similar studies on automated sleep staging. It is publicly available, which is conducive to expanding access to sleep-related analysis, especially those associated with neurological or psychiatric disorders.

## 1. Introduction

The sleep staging based on overnight polysomnography (PSG) plays an important role in diagnosing and treating the sleep disorders and performing research related to psychiatric diseases (Baglioni et al., 2016; Freeman et al., 2020). As the manual sleep staging process is laborious, tedious, and time-consuming, the inter-rater and intra-rater reliability are prone to subjective uncertainty (Rosenberg and Van Hout, 2014). The automatic scoring or at least automatic assistance of sleep staging has been studied for several decades and has attracted considerable attention. The traditional machine learning for performing PSG analysis usually employs hand-tuned feature combinations (Diykh et al., 2016; Pillay et al., 2018; Sharma et al., 2018; Vallat and Walker, 2021). Due to an increase in the computing power, the dependence on the hand-tuned features has decreased rapidly for performing physiological signal analysis (Craik et al., 2019; Buongiorno et al., 2020). Significant progress has been reported in sleep staging based on deep learning.

The performance of deep learning-based methods approaches or exceeds human performance in terms of the implementation of PSG sleep staging. Patanaik et al. (2018) developed a scoring framework with accuracy comparable to that of human expert raters and validated it on data including Parkinson’s disease patients. Stephansen et al. (2018) achieved reliably scoring down to 5 second epoch sleep and performed better than any individual scorer. According to the staging rules, Qu et al. (2020) refinedly optimized the network structure to further improve the staging performance. Perslev et al. (2021) developed a high-resolution sleep staging model on large-scale multi-cohort data, achieving greater versatility on unseen datasets. Bakker et al. (2022) compared their automated sleep staging algorithms with the results of 6–12 human scorers, revealing an inherent relationship between the probabilities of sleep stages obtained by the two methods, thereby providing encouraging support for the potential of automated sleep staging. In a recent report, researchers recognized the importance of differentiating confusing stages and optimized their methods accordingly, providing a theoretical basis that partially supports our subsequent research method (Phyo et al., 2022). However, there still exist various issues in this implementation that should be properly addressed. For instance, a well-known problem is the low agreement of stage N1 scoring.

The N1 sleep has short duration, less distinct features, and strong dependence on its pre- and post-epoch relations. In addition, it has the lowest agreement rate (63.0%) among different human scorers (Rosenberg and Van Hout, 2014; Younes et al., 2018). During the training process of sleep staging models, these characteristics make it more challenging to train N1 epochs than others. Recognizing stage N1 remains one of the biggest challenges when developing automatic sleep staging models. In some research areas, lower accuracy of stage N1 affects the results to a small extent. However, as the proportion of N1 sleep of the subject increases, it damages the agreement and applicability of scoring results. Studies have shown that N1 sleep is more prevalent during certain conditions or diseases. The frequent awakenings in sleep disorders, such as insomnia and sleep fragmentation lead to an increase in N1 sleep (Merica, 1998; Baglioni et al., 2014; Wei et al., 2017). Specific people, such as the elderly, alcoholics, and individuals suffering from chronic pain, were reported to have higher amount of N1 sleep (Ohayon et al., 2004; Gulia and Kumar, 2018; Mathias et al., 2018; Koob and Colrain, 2020). Please note that this feature is more prominent in patients with neurological or psychiatric disorders. Therefore, in various studies, N3 replaces N1 as the least frequent stage of sleep in such patient groups. The Schizophrenia patients are reported to have higher rates of N1 in sleep as compared to the healthy controls ranging from 2.2 to 15.8% (Göder et al., 2004; Yang and Winkelman, 2006; Sarkar et al., 2010; Chan et al., 2017). The Alzheimer’s disease (AD) significantly increases N1 sleep, i.e., more than 30% of the total sleep, due to the derangements of sleep–wake cycle regulatory pathways (Liguori et al., 2014). N1 sleep has also been reported as an influential node in sleep research focused on moderate depression (Elovainio et al., 2019). Therefore, accurate scoring stage N1 is of great significance for hospital-based research or patients with certain diseases.

The existing automated sleep stage scoring algorithms have not received sufficient attention due to the low impact of stage N1. It is noteworthy that most of the algorithms learn by using the public datasets derived from the population-based studies and not the disease-specific datasets. The proportion of stage N1 during the overnight PSG of healthy subjects is small. This does not affect the overall performance of the model. Some researchers have used oversampling or feature over-expression for improving the N1 accuracy, however, this comes at an expense of identifying other stages (Supratak et al., 2017; Chambon et al., 2018; Johnson and Khoshgoftaar, 2019; Zhang et al., 2023). The diseased individuals have more complex sleep structures, and it is not clear whether these algorithms can be effectively utilized for overcoming diseases or in hospital-based studies.

In this work, we present an automatic sleep staging method and apply it to sleep PSG. The purpose of the proposed method is to address the identification difficulties of N1 sleep due to its low-resource in sleep staging, and accomplish accurate scoring tasks in other stages. The major contributions of this work are presented below.

1). The proposed model achieves high overall performance while realizing an N1 accuracy with the level of human scorers.
2). We propose a gradual transitional training scheme based on a two-branch trade-off network for coping with the feature relationship between one epoch and its context. This reduces the risk of class rebalancing in network training.
3). In contrast to most of the existing work, the generalization performance of the proposed model is evaluated in a mixed cohort test and in a cross-dataset test.
4). We also extend the model for group benchmarking of psychiatric disorders to highlight its advantages for patients.

## 2. Materials and methods

### 2.1. Study datasets

In this work, we use seven publicly available datasets in five cohorts. The model is first trained and evaluated on a large-scale public dataset, i.e., the sleep heart health study (SHHS) database, which is approved by the National Sleep Research Resource (Quan et al., 1997; Zhang et al., 2018). Once the model is trained, the data from the other four cohorts, including Cleveland Children’s Sleep and Health Study (CCSHS, n=515 ) (Rosen et al., 2003), Study of Osteoporotic Fractures (SOF, n=453 ) (Spira et al., 2008), Cleveland Family Study (CFS, n=730 ) (Redline et al., 1995), and MrOS Sleep Study (MrOS1, n=2905 ; MrOS2, n=1026 ) (Blackwell et al., 2011) are adopted for two forms of multi-cohort evaluation.

The SHHS cohort comprises two rounds of PSG recordings named SHHS1 (*n* = 5,793) and SHHS2 (*n* = 2,651). SHHS1 is the largest dataset in this study, which is first used for algorithm development and comparison. SHHS2 comprises second acquisition time points for a subset of SHHS1 subjects and is only used along with the other cohorts for performing multi-cohort evaluation to prevent the potential self-reporting bias. CCSHS is a pediatric cohort that differs significantly from other cohorts in terms of age distribution of the subjects. SOF and MrOS are gender-specific cohorts that include older subjects. CFS is a large family-based study cohort with a wider age range of subjects, and its fifth visit of the PSG is employed in this work. The stages of PSGs in the aforementioned datasets are scored and organized by using the prevalent AASM guidelines (Iber et al., 2007).

The demographic and general sleep characteristics for these datasets are presented in Table 1.

**Table 1:** Demographics and general sleep characteristics of datasets.

	SHHS1	SHHS2	CCSHS	SOF	CFS	MrOS1	MrOS2
*N* (female)	5,793 (3033)	2,651 (1425)	515 (255)	453 (453)	730 (401)	2,905 (0)	1,026 (0)
Age, years	63.1 ± 11.2	62.4 ± 10.5	17.7 ± 0.44	82.8 ± 3.13	41.4 ± 19.4	76.4 ± 5.5	81.0 ± 4.4
AHI, h	17.9 ± 16.1	18.4 ± 16.4	1.8 ± 5.1	16.3 ± 13.9	12.5 ± 17.0	–	–
TRT, min	1012.1 ± 74.7	1204.3 ± 137.0	1342.4 ± 95.8	1194.5 ± 293.0	1186.8 ± 108.1	1296.7 ± 205.9	1569.3 ± 349.9
Wake, %	28.7 ± 12.3	37.4 ± 11.6	30.5 ± 10.4	39.7 ± 15.2	36.8 ± 12.7	44.5 ± 11.7	54.5 ± 13.3
N1, %	3.7 ± 2.6	3.5 ± 2.9	2.8 ± 1.6	3.0 ± 1.9	3.0 ± 2.3	3.6 ± 2.1	5.3 ± 3.7
N2, %	41.0 ± 11.4	36.2 ± 9.5	36.2 ± 7.4	33.6 ± 11.2	35.5 ± 10.3	34.9 ± 8.9	28.5 ± 10.1
N3, %	12.6 ± 8.8	9.9 ± 7.2	16.0 ± 5.9	12.5 ± 8.6	13.0 ± 9.3	6.3 ± 5.3	3.1 ± 3.5
REM, %	14.0 ± 5.8	13.0 ± 5.1	14.5 ± 4.5	11.2 ± 5.5	11.6 ± 5.5	10.7 ± 4.7	8.6 ± 4.2

N: number of recordings, AHI: apnea/hypopnea index, TRT: total recording time.

### 2.2. Data preparation

The raw time series comprising five channels (EEG: C3 and C4, EOG: left and right, EMG: chin) are filtered (EEG/EOG: High Pass 0.3 Hz/Low Pass 35 Hz, EMG: High Pass 10 Hz), clipped (-500 ~ 500 μV), and resampled (125 Hz). Then, these signals are used as the training inputs without further pre-processing or artifact removal, as pre-processing does not improve the performance of the models significantly. Instead, more diverse data is commonly beneficial for enhancing the model’s robustness (Nazaré et al., 2018; Zhang et al., 2019; Olesen et al., 2020).

### 2.3. Network architecture

**Figure 1:**
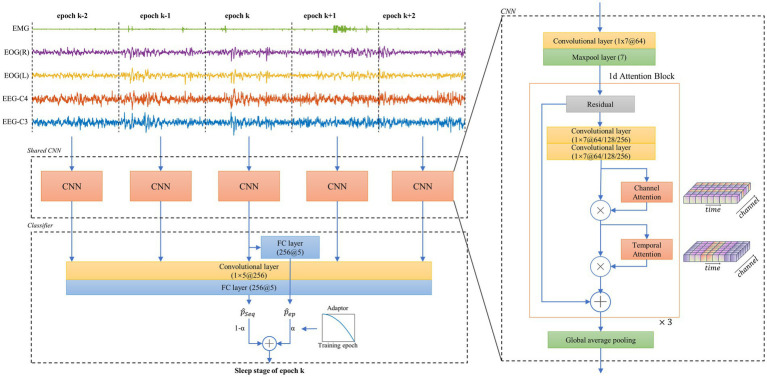
Overall architecture of the neural network and its feature extractor. The left part shows the workflow of the proposed neural network. A 30-s epoch k of multi-channel polysomnography (PSG) and its two consecutive epochs before and after are simultaneously input to the feature extractor. The proposed two-branch classifier then computes the sleep/wake stage of epoch k; The right part shows a basic unit of the shared CNN structure, which includes the proposed 1d attention block. CNN = convolutional neural network.

An end-to-end network is implemented for sleep staging. This network consists of two parts, i.e., a CNN comprising 1-d attention residual block for feature extraction and a classifier that not only considers the single epoch features but also looks back and forward through the consecutive epochs. An overview of this neural network architecture and algorithm flowchart for sleep epoch k is presented in Figure 1 (left). Three cascaded residual blocks, presented as 1-d attention block, are designed to construct this CNN. In each block, two soft attention layers, namely channel attention layer and temporal attention layer, are added behind the output of the convolutional layer. In each block, there are two one-dimensional convolutional layers with 1 × 7 kernels used for generating the intermediate feature, i.e., $F\in {\mathbb{R}}^{C\times T}$ , where C and T represent the feature number of the channel and the temporal dimensions. Afterwards, the attention modules are applied on F for emphasizing and suppressing the meaningful features in independent dimensions. In the channel dimension, as each channel is considered a feature detector, the relationships among channels are used to generate a channel attention map. In order to achieve this, a multi-layer preceptor ( MLP ) with squeeze-and-excitation structure is implemented to extract the relationships from the global pooled features (Hu et al., 2020; Huang et al., 2022). This module follows the channel attention scheme used in CBAM (Woo et al., 2018). The final channel attention map ( $M_{C}$ ) is computed as follows:


(1) $$M_{C}=Softmax\left(MLP\left(AvgPool\left(F\right)\right)+MLP\left(MaxPool\left(F\right)\right)\right)$$

where, F denotes the input feature and is pooled in its temporal axis.

In the temporal dimension, a max-pooling operation along the channel axis is implemented to find the most attended waveform characteristics among all channels. A cascaded SoftMax layer is used to prevent the excessive gains caused by the temporal attention map. The proposed temporal attention map ( $M_{T}$ ) is expressed as follows:


(2) $$\begin{array}{c} M_{T}=1+Softmax\left(MaxPool\left(F\right)\right) \end{array}$$

where, F denotes the input feature and is pooled in its channel axis.

Figure 1 (right) shows that $M_{C}$ and $M_{T}$ are sequentially arranged after convolutional features to form a 1-d attention block, which is used to separately solve the *what* and *where* problems in attention tasks (Woo et al., 2018). A global average pooling layer is connected to these three cascaded blocks for extracting the features with a dimension of 256.

The classifier contains two separate branches with fully connected networks, namely epoch learning branch (ELB) and sequential learning branch (SLB). The ELB uses 256 features of epoch k as an input for learning the universal patterns. The SLB uses 256 × 5 features of N consecutive epochs before and after epoch k for sequential modeling. In this work, N is determined to be 2 based on the pre-experiment, since a larger N leads to less than 0.3% improvement in accuracy. The two branches separately output the probabilities $\hat{p}$ for five stages ( ${\hat{p}}_{ep}$ for ELB and ${\hat{p}}_{seq}$ for SLB). The main difference between the two branches lies in their contextual perspectives and the training difficulty determined by the number of trainable parameters. We also present a novel transitive training strategy to merge ${\hat{p}}_{ep}$ and ${\hat{p}}_{seq}$ . Finally, the predicted sleep/wake stage of epoch k is obtained by a weighted average of the two branches.

### 2.4. Transitive training strategy and model training

The recognition of stage N1 is considered a low-resource problem (Morfi and Stowell, 2018). There are three reasons why stage N1 identification is challenging as we examined. Firstly, the N1 epochs are underrepresented (the long-tail problem), which accounts for a small proportion in sleep/wake stages. Secondly, N1 is the only stage with almost no characteristic grapho-elements (Deng et al., 2019). The features of N1 are more easily confused (such as the variability in EOG and EMG, the possible presence of alpha rhythms similar to those observed during Wake, and the occurrence of V-waves with a certain probability), and for this reason we utilized the low occurrence of N1 to allow the initial training to be less focus on N1 features to reduce the cost. Finally, the decision of N1 is more significantly context-dependent, especially people who generated little or no α activity (Danker-Hopfe et al., 2009). Therefore, the use of simple model rebalancing may lead to the learning of more confounding features, which can potentially harm the overall performance of the model.

We propose a novel transitive training strategy for minimizing the impact of rebalancing N1 on the overall performance. In order to accomplish this, in the training process, a decreasing factor α is automatically generated in the training phase, and the classification loss L is calculated as follows:


(3) $$\begin{array}{c} L=\alpha L_{ep}+\left(1-\alpha \right)L_{seq} \end{array}$$

where, $L_{ep}$ denotes the unweighted cross-entropy of ${\hat{p}}_{ep}$ and y, and $L_{seq}$ denotes the weighted cross-entropy of ${\hat{p}}_{seq}$ and y. The weights of each class are equally proportional to the inverse of the number of samples in that class.

During the training process, the learning attention gradually shifts from ELB to SLB. This means that the model first learns the universal patterns with less influence of the weak characteristics, and then gradually improves the performance of the minority classes under these universal patterns and sequential information from context. Here the universal pattern represents the features from original signals learned from the actual data distribution. Notably, $L_{seq}$ is set to back-propagate only in the sequential learning branch and does not affect the CNN parameters, thereby making the training difficulties of both branches comparable. Inspired by the cumulative learning (Zhou et al., 2020), α is calculated as follows:


(4) $$\begin{array}{c} \alpha =1-{\left(T/T_{\max}\right)}^{2} \end{array}$$

where, $T_{\max}$ denotes the total number of training epochs and T denotes the current epoch. The value of α during the training process are shown in Supplementary Figure S1A.

### 2.5. Evaluation metrics

We use accuracy, F1 score, and Cohen’s kappa for assessing the performance of the model. Specifically, we use accuracy to evaluate the overall performance of the model scoring, Cohen’s kappa to assess the inter-rater agreement between the manual and automatic scoring, macro-averaged F1 (MF1) to assess the impact of class imbalance on model’s performance and for selecting the best model. The accuracies for each class are presented, which are calculated as the proportion of the samples detected correctly for each class (recall). In order to present additional test details, we also calculate the confusion matrix, in which each element (i,j). represents the empirical probability, i.e., class i is predicted to be class j.

If not specified, the metrics are reported through *by-epoch* statistics (each epoch is an independent sample in the dataset). Furthermore, boxplots in this paper demonstrate the metrics through *by-record* statistics (for each PSG separately).

### 2.6. Experimental setups

In this study, we conducted four experiments as follows:

A. We first develop the proposed algorithm based on 5,793 subjects from SHHS1 dataset to determine the hyperparameters of the model and compare it with other algorithms. The PSG records are randomly split into training, validation, and test sets by ratios of 80, 10, and 10%, respectively. As shown in Table 2, there are no significant differences in demographics or class proportions for the three split subsets. The model developed on training set and achieving the highest MF1 on validation set is used for evaluation on test set. We conduct moderator analyses on SHHS1 data and investigate how different subgroups, training methods, and α settings influence the results.
B. The model training and testing are performed on mixed cohorts. We adopt 14,118 PSGs from seven datasets (SHHS1, SHHS2, CCSHS, SOF, CFS, MrOS1, MrOS2) in five cohorts for conducting a mixed-cohort evaluation. Please note that we use the same experimental settings and hyperparameters as experiment (A).
C. We use leave-one-set-out validation approach to estimate the generalizability of the proposed algorithm. The algorithm is applied once for each dataset, where all other datasets are used as training/validation sets and the selected dataset or cohort is used as the test set. For performing this validation, the model is trained several times with the same hyperparameters as discussed in experiment (A).
D. We accomplish a performance comparison of disease-related sleep staging. A study with similar cohort size and similar experiments as presented in this work is introduced as a benchmark (Olesen et al., 2020). Please note that the CFS is selected as the test set due to its significant variability in terms of N1 sleep. In addition, it is not used as the training set in the trained benchmark model. We evaluate the two algorithms using two subgroups, divided according to whether the subjects had significant neurological or psychiatric disease.

**Table 2:** Overview of SHHS1 dataset splitting in Experiment A.

SHHS1	M/F, %	Age	AHI	BMI	Wake epoch	N1 epoch	N2 epoch	N3 epoch	REM epoch
Training set	47/53	63.1 ± 11.3	17.8 ± 16.0	28.1 ± 5.1	1,335,339 (28.6%)	174,749 (3.7%)	1,915,026 (41.0%)	589,641 (12.6%)	652,788 (14.0%)
Validation set	48/52	62.9 ± 11.5	18.9 ± 17.3	28.2 ± 5.0	166,318 (28.7%)	20,415 (3.5%)	237,214 (40.9%)	74,517 (12.8%)	81,905 (14.1%)
Test set	48/52	63.6 ± 10.7	18.2 ± 16.0	28.1 ± 5.0	166,906 (28.5%)	21,817 (3.7%)	240,713 (41.1%)	74,543 (12.7%)	81,339 (13.9%)

Age, AHI, and BMI distributions were not significantly different among the three split subsets, as determined by one-way ANOVA. M/F: male/female, AHI: apnea/hypopnea index, BMI: body mass index.

During the training process, a mini-batch size of 200 is used. The initial learning rate is set to 0.001 for Adam optimizer (Kingma and Ba, 2014). Each model is trained for 140,000 iterations with a 10% learning rate decay after 70% of the training process is completed.

## 3. Results

### 3.1. Model training and testing on SHHS1 cohort

In each data input operation during the training process, a thirty-second epoch of PSG and two succeeding and preceding epochs are simultaneously fed into the network. In Supplementary Figures S1B,C, we show the training curves of the model in experiment (A), where each epoch contains 3,500 mini-batch training iterations. After approximately thirty training epochs, the curves of the training loss, validation loss, and MF1 begin to plateau. However, the training MF1 curve decreases significantly. Supplementary Figures S1D–F illustrates that predicting the sleep/wake stages with the average output of two branches is better instead of only using one branch in our experiment.

**Figure 2:**
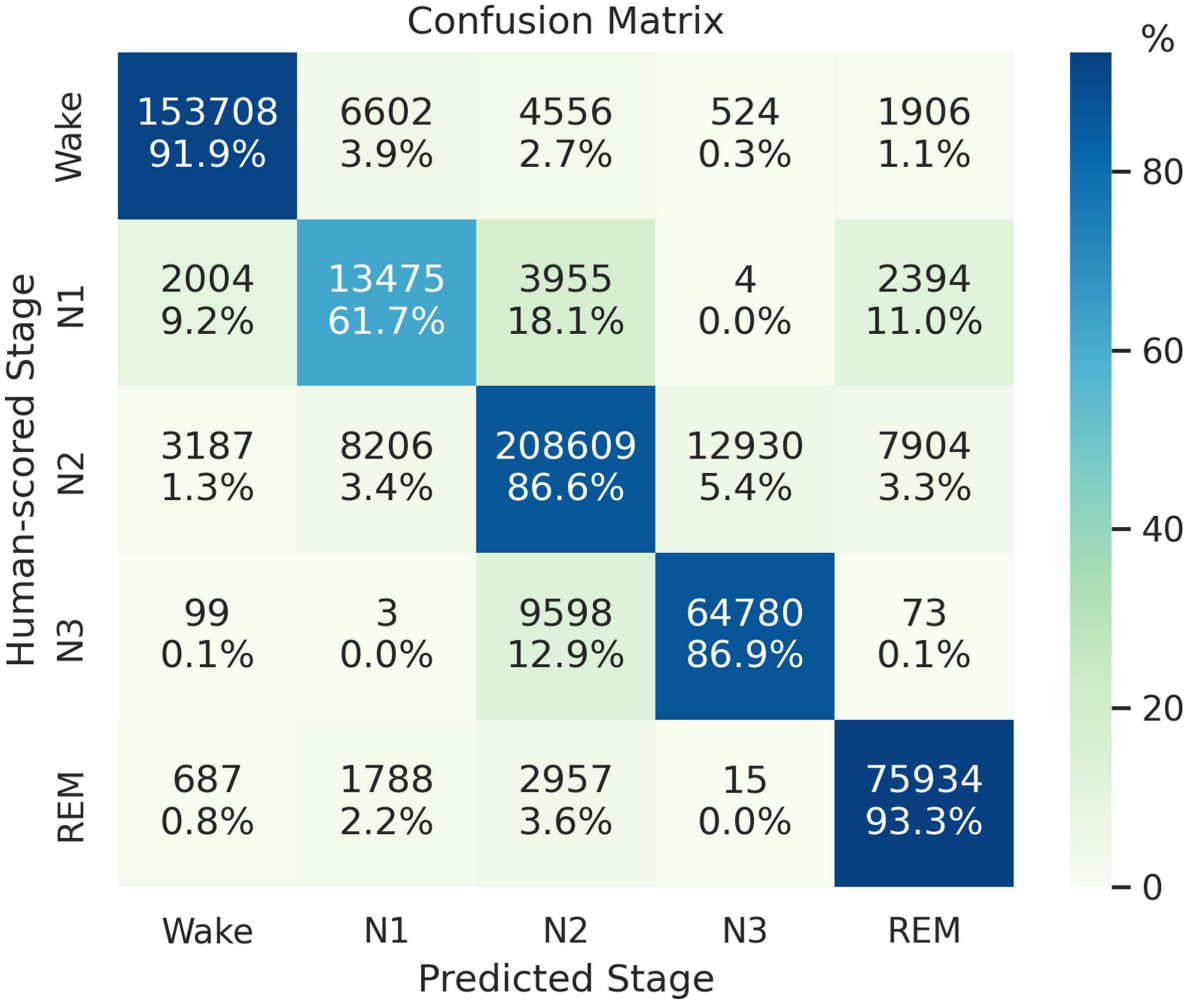
Confusion matrix of the classification result on the SHHS1 test set. Each row represents the instances with human-scored labels in this matrix, and each column represents instances with machine-scored labels.

On the SHHS1 test set, the model achieves an accuracy of 88.16%, Cohen’s kappa of 0.836, and MF1 score of 0.818. The confusion matrix presented in Figure 2 shows that the proposed network correctly classifies 91.9, 61.7, 86.6, 86.9, and 93.3% of Wake, N1, N2, N3, and REM stages in the test set, respectively. Table 3 presents a comparison of proposed method and other automatic sleep staging approaches developed and evaluated on SHHS datasets (Sors et al., 2018; Zhang et al., 2019; Fernandez-Blanco et al., 2020; Seo et al., 2020; Xu et al., 2020, 2022; Eldele et al., 2021; Pathak et al., 2021; Vallat and Walker, 2021; Sharma et al., 2022; Zhao et al., 2022; Zhang et al., 2023). Most reported values represent the best performance reported in their original publications, and we added a few missing metrics based on their reported results. It should be noted that the comparison is approximate due to variations in data splitting or channel selection across the compared methods. Furthermore, there are several other recent studies not included in Table 3 due to differences in their datasets or experimental designs (Jia et al., 2022; Phyo et al., 2022; Zhao et al., 2022). These studies showed agreements ranging from 86.4 to 87.7% in terms of overall accuracy, with the best reported results achieved under their respective study designs, but also demonstrated a need for further improvement in N1 classifications.

**Table 3:** Model performance compared to other studies on SHHS dataset.

Method	Dataset	Record	Channel	Train/(Validation)/Test	Wake Acc (%)	N1 Acc (%)	N2 Acc (%)	N3 Acc (%)	REM Acc (%)	Overall Acc (%)	Cohen’s Kappa	MF1
(Sors et al., 2018)	SHHS1	5,728	1EEG	0.5/0.2/0.3	91	35	89	85	86	87	0.81	0.78
IITNet (Seo et al., 2020)	SHHS1	5,791	1EEG	0.5/0.2/0.3	92	42	88	85	87	86.7	0.81	–
(Eldele et al., 2021)	SHHS1 SHHS2	329	1EEG	20 folds	88.3	46.3	88.7	**87.6**	87.4	86.6	0.81	0.797
SleepContextNet (Zhao et al., 2022)	SHHS1	329	1EEG	20folds	89.6	52.0	87.6	84.3	89.6	86.4	0.81	0.805
(Fernandez-Blanco et al., 2020)	SHHS1	5,804	2EEG	0.7/0.1/0.2	91.2	22.1	**91.6**	82.1	82.8	85.2	0.79	0.76
(Xu et al., 2020)	SHHS1 SHHS2	8,444	2EEG, 2EOG, 1EMG	5793/2651	**94.2**	44.2	88.6	77.5	89.6	87.6	0.825	–
(Zhang et al., 2019) imbalanced	SHHS1	5,793	2EEG, 2EOG, 1EMG	0.9/0.1	92	37	91	77	88	87	0.82	–
(Zhang et al., 2019) balanced	SHHS1	5,793	2EEG, 2EOG, 1EMG	0.9/0.1	91	46	89	77	88	86	0.82	0.81
STQS (Pathak et al., 2021)	SHHS1	5,793	2EEG, 2EOG, 1EMG	0.81/0.09/0.1	92.5	40.3	84.4	76.0	89.1	84.9	0.765	0.79
(Vallat and Walker, 2021)^a^	SHHS1	689	2EEG, 2EOG, 1EMG	590/99	91	44	86	81	89	-	-	–
(Xu et al., 2022)	SHHS1 SHHS2	8,444	2EEG, 2EOG, 1EMG	5793/2651	92.35	24.39	89.10	79.18	87.49	86.85	0.8115	–
(Sharma et al., 2022)	SHHS1	5,791	2EEG, 2EOG, 1EMG	0.82/0.02/0.1	93	12	90	77	75	84.30	0.7746	-
(Zhang et al., 2023)^b^	SHHS1	5,793	2EEG, 2EOG, 1EMG	0.8/0.1/0.1	92.5	42.1	90.2	79.0	91.8	87.88	0.829	0.803
Proposed	SHHS1	5,793	2EEG, 2EOG, 1EMG	0.8/0.1/0.1	91.9	**61.7**	86.6	86.9	**93.3**	**88.16**	**0.836**	**0.818**

The bold values indicate the highest values within each column.

a Model in this study was not only trained on the SHHS dataset.

b Reproduced and fine-tuned the model using the code and hyperparameters from the original authors.

In order to validate the effectiveness of the proposed two-branch rebalancing strategy, we train the network after removing the ELB branch with a no rebalancing strategy and a weighted rebalancing strategy with weights being inversely proportional to the scale of each class (Goshtasbi et al., 2022). The performance metrics of the three models are presented in Supplementary Figure S2. Please note that each confusion matrix is significantly correlated with that of the human raters presented in Rosenberg and Van Hout (2014) with Pearson correlation coefficients of 0.975 (no rebalancing), 0.984 (proposed), and 0.975 (weighted rebalancing). In summary, the proposed algorithm serves as a trade-off between the other two methods, and the resulting model scored more closely to human scoring in terms of the correctness of each class. More specifically, as compared to no rebalancing strategy, the weighted strategy sacrificed the agreement of 23,256 (9.7%) N2 epochs in exchange for 7,895 (36%) N1 epochs, and the proposed strategy sacrificed the agreement of 5,476 (2.3%) N2 epochs in exchange for 4,325 (25%) N1 epochs.

### 3.2. Moderator analyses

The inter-rater agreement of the transition segment staging is generally lower as compared to the stable segments (Rosenberg and Van Hout, 2013). We evaluate the performance of the proposed model for stable and transitional epochs in the test set. An epoch is defined as a stable epoch if its label is the same as its preceding and following epochs; otherwise it is regarded as the transitional epoch. The proposed model is used to separately score the stable epochs (*n* = 472,658) and the transitional epochs (*n* = 113,240). Two confusion matrices are presented in Supplementary Figure S3. The performance of the proposed model during stable epochs (accuracy = 91.62%, Cohen’s kappa = 0.882, MF1 = 0.813) is much higher as compared to the transitional epochs (accuracy = 73.69%, Cohen’s kappa = 0.649, MF1 = 0.724).

We also investigate the effect of different settings of α on the testing results. Supplementary Figure S4 shows the test results of stable epochs and transitional epochs under different values of α. It is noteworthy that a higher α helps to further increase the accuracy and Cohen’s kappa of the results. This effect is more pronounced in testing stable epochs as compared to testing transitional epochs.

This proposed model is additionally evaluated based on the subgroups of SHHS1 test set that contains no (Apnea-Hypopnea Index, AHI <5), mild to moderate (5 ≤ AHI ≤30), and severe (AHI >30) obstructive sleep apnea (OSA). Similar to the experiment discussed in the previous section, the PSG records in three subgroups are directly input in the trained model without additional finetuning within each group. This model achieves an accuracy of 89.33% (no OSA), 88.26% (mild to moderate OSA), and 86.58% (severe OSA); Cohen’s kappa of 0.854 (no OSA), 0.837 (mild to moderate OSA), and 0.809 (severe OSA); the MF1 score of 0.832 (no OSA), 0.818 (mild to moderate OSA) and 0.801 (severe OSA). Three confusion matrices are presented in Supplementary Figure S5. The three subgroups have minor differences in the performance curves at different values of α, as shown in Supplementary Figure S6.

### 3.3. Model training and testing on mixed cohorts

**Figure 3:**
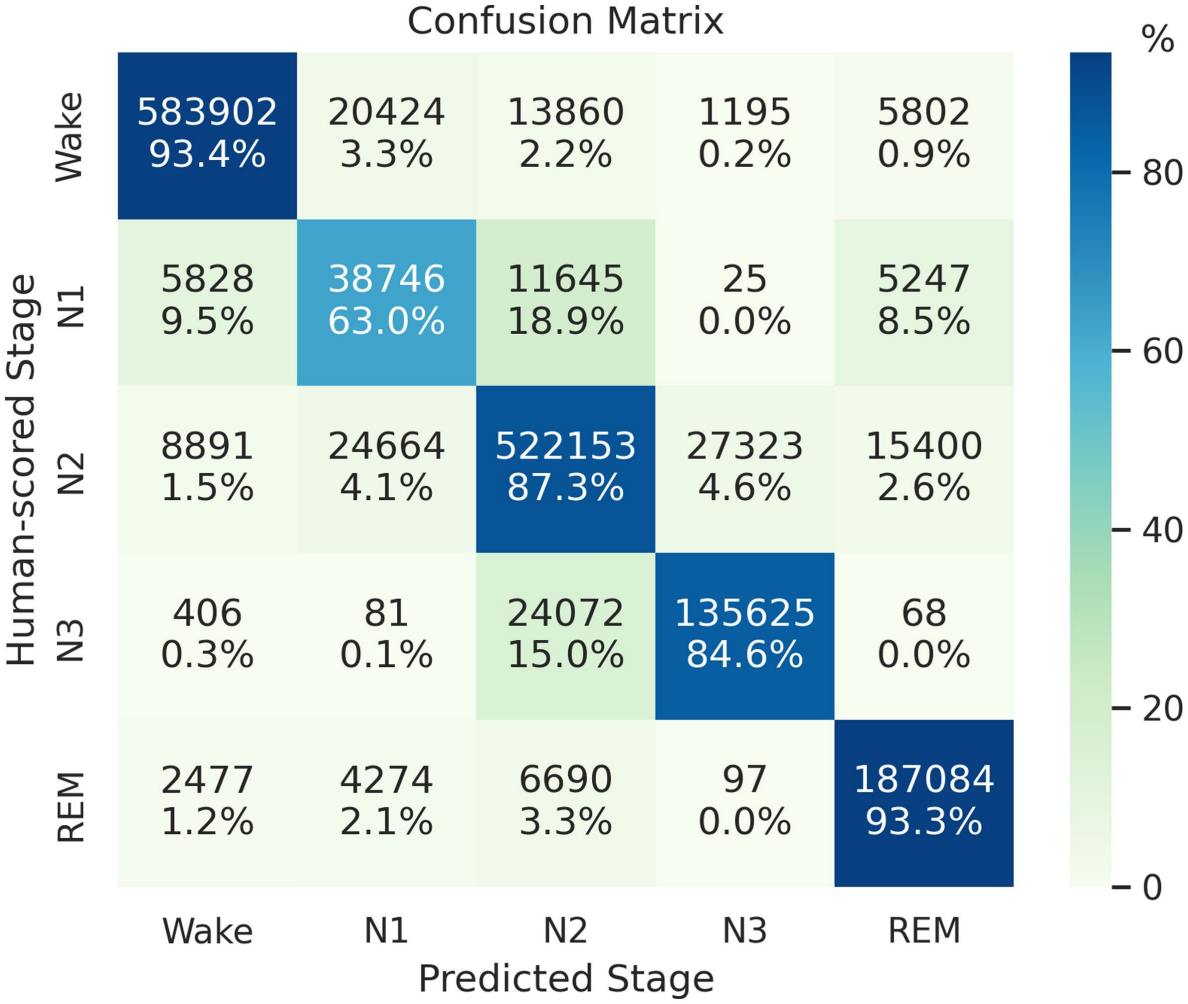
Confusion matrix of the classification result on the mixed-cohort test set. Each row represents the instances with human-scored labels in this matrix, and each column represents instances with machine-scored labels.

We train and test the proposed model by using 14,118 PSG records obtained from five cohorts. The proposed model predicted 1,645,979 epochs of the test set with an accuracy of 89.16%, Cohen’s kappa of 0.846, and MF1 score of 0.819. Figure 3 presents the model’s classification confusion matrix in this test, which is comparable to or better than the results evaluated in SHHS1.

**Figure 4:**
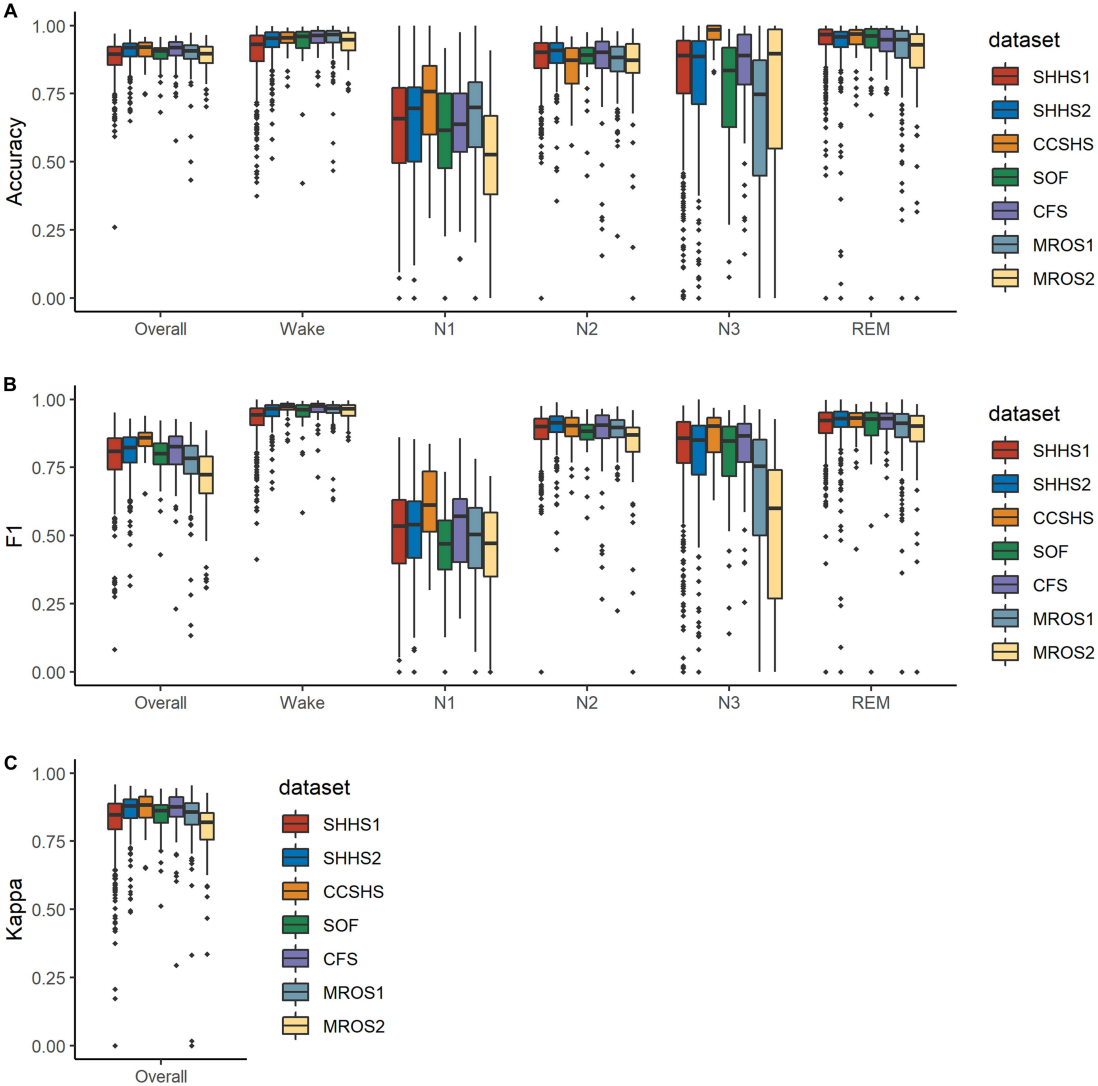
Boxplots illustrating the distributions of the metrics on mixed-cohorts test subjects. In **(A,B)**, metrics for each class are shown along with their overall metrics, where the overall F1 is calculated as macro-F1. **(C)** shows Cohen’s kappa values for the five classes. Note that some PSG recordings were manually scored with one or more stages having a count of zero and are not included in the corresponding accuracy calculation.

The distribution of testing metrics for PSG recordings from each dataset is presented in Figure 4. Please note that the performance of this model varies significantly across subjects in N1 and N3 stages and is broadly similar across datasets.

### 3.4. Cross-dataset validation

In order to investigate the performance of the proposed algorithm on unseen data, we adopt leave-one-set-out cross validation for evaluating the model. We separately hold out one dataset for testing and use the combined data from other datasets for training the model. Considering the existence of two visits for the same subjects (SHHS, MrOS), and the need to test the unseen subjects in practical application of the model, we left the two cohorts separate for performing additional validation. The cross-dataset validation results are presented in Table 4. In short, the performance of the proposed model varies little for different unseen datasets. Leaving out one cohort performed worse as compared to leaving out only one visit of it.

**Table 4:** Cross-dataset validation results of proposed model.

Dataset^**a**^		Number of test epoch	Wake Acc %	N1 Acc %	N2 Acc %	N3 Acc %	REM Acc %	Overall Acc %	Cohen’s Kappa	MF1
Test	Training									
1,2	3,4,5,6,7	9,019,609	93	56	86	70	86	85.35	0.794	0.772
1	2,3,4,5,6,7	5,839,022	*92*	59	86	71	94	85.94	0.804	0.789
2	1,3,4,5,6,7	3,180,587	93	60	88	91	93	89.78	0.856	0.822
3	1,2,4,5,6,7	687,960	94	69	87	93	93	90.36	0.869	0.845
4	1,2,3,5,6,7	538,071	96	62	85	82	93	89.13	0.844	0.809
5	1,2,3,4,6,7	862,228	95	61	85	88	92	89.04	0.847	0.817
6,7	1,2,3,4,5	5,358,310	95	60	81	86	89	87.95	0.817	0.778
6	1,2,3,4,5,7	3,753,884	95	68	82	79	92	88.25	0.826	0.789
7	1,2,3,4,5,6	1,604,426	94	53	87	78	89	89.34	0.825	0.779
Leave 1–7 arithmetic mean			94.1	61.7	85.7	83.1	92.3	88.834	0.839	0.807
Leave 1–7 weighted mean			93.4	61.3	85.5	79.5	92.7	87.991	0.827	0.799

a Datasets are represented by numbers: 1 = SHHS1, 2 = SHHS2, 3 = CCSHS, 4 = SOF, 5 = CFS, 6 = MrOS1, 7 = MrOS2.

### 3.5. Benchmark comparison in disease-related sleep

We state a participant as “having significant neuro-logical or psychiatric disease” when they had at least one neurological or psychiatric diagnosis in the medical record (See the footnote of Table 5 for details). The CFS is divided into two subgroups based on the aforementioned standards, the psychiatric group (PSY), and the healthy control group (HC). As presented in Supplementary Figure S7, significant differences are observed for the stage proportions in sleep between PSY and HC groups. Please note that the PSY group has more light sleep as compared to HC group. For performing comparative evaluation, the benchmark algorithm (Olesen et al., 2020) uses the trained model available at https://github.com/neergaard/deep-sleep-pytorch, and ours used the best model in the leave-CFS-out evaluation. Note that the input sequence duration of the benchmark model is 5 min, while ours is 2.5 min.

**Table 5:** Benchmark comparison results on two CFS subgroups, divided by psychiatric disorders.

Method	Subgroup	Record	Wake Acc	N1 Acc	N2 Acc	N3 Acc	REM Acc	Overall Acc	Cohen’s Kappa	MF1
(Olesen et al., 2020)	HC	549	94.7%	41.0%	91.2%	69.8%	86.7%	87.5%	0.822	0.785
	PSY	181	95.6%	38.7%^*^	90.6%	66.8%^*^	88.6%	87.9%	0.824	0.781
Proposed	HC	549	95.0%	61.1%	84.4%	88.8%	92.0%	89.1%	0.847	0.818
	PSY	181	94.7%	62.4%	85.3%	85.7%^*^	91.7%	88.9%	0.841	0.813

Groupings were based on psychiatric-related medical history records from the CFS publicly available PSG dictionaries. The variable names are psydiag, strodiag, adddiag, anxdiag, depdiag, cerebdisease, parkdiag, dementia, and mscldiag in their version 0.5.0 archive. HC = healthy control. PSY = Psychiatric Disorders. Significance (*): *p* < 0.05, independent t-test for HC vs. PSY mean difference.

Table 5 demonstrates that two algorithms have significantly different predictive propensities. As compared with the HC group, both algorithms achieve significantly worse N3 accuracy and comparable overall performance on PSY group. The benchmark algorithm more accurately predicted Wake, REM, and worse predicted light sleep stages (N1, N2) for the PSY group, while the proposed algorithm did the opposite. For both groups, the proposed model obtains lower N2 accuracy but achieved higher N1, N3, REM and overall performance as compared to the benchmark algorithm.

## 4. Discussion

A challenge with sleep staging in previous studies is that they tend to suffer from lower agreement of stage N1, as it only occupies about 5% of healthy individuals’ overnight sleep. In addition, its recognition is more likely confused with other stages (Basner et al., 2008; Suzuki et al., 2019). The low N1 accuracy usually has a minor impact on the overall agreement of the scorers. However, certain diseases result in more N1 sleep. Therefore, more accurate stage N1 identification is necessary for specific studies. Balancing or oversampling has been used in other literature. However, increasing N1 accuracy leads to decreasing the overall performance, which is difficult to address (Chambon et al., 2018; Zhang et al., 2019). In this work, we propose a neural network-based automated sleep staging model for polysomnography to address the low-resource problem of N1 sleep staging, which is characterized by limited samples and ambiguous features.

We observe that preprocessing steps such as denoising, detrending, and normalization have negligible impact on the model’s performance. Instead, using a large-scale dataset enables us to develop a robust model that can handle signal variability. To enhance feature representation, we implement an attention mechanism based on 1-D feature layers. This approach enables a CNN model with only three residual blocks to effectively extract PSG features without compromising classification results. We also explore the ablatively use of recurrent neural networks and transformers for classification and observe performance metric increases by −0.2% ~ 0.7% for testing based on SHHS1, but by −0.5% ~ 0.0% for testing based on mixed cohort.

Scoring accuracy of N1, a minority and challenging class, is significantly improved in this study and has reached the level of human scorers (Rosenberg and Van Hout, 2013, 2014). Considering that there are fewer distinctive features in stage N1, and the decision of N1 epochs is more dependent on the consecutive epochs before and after them, the proposed ELB without class rebalance is first trained to extract the universal features. In the later training process, the proposed SLB with class rebalancing gradually dominates the training. The training focus of the two branches is gradually adjusted by a decreasing adapter. This strategy enables the feature extractor to learn from the majority class first, which can prevent the model from struggling with difficult feature learning. It then enhances the recognition of stage N1 by incorporating contextual information and minimizing the risk of interference from confused features. This training strategy leverages the N1 scoring experience of human scorers by relying more on contextual features and fewer epoch features, thereby minimizing the risk of universal patterns interfering with N1 epoch recognition during training.

During the testing phase, the two-branch trade-off parameter α is fixed to 0.5 as the assumption that two branches are equally important. When changing the testing α with grid search, we observe that the overall accuracy improves by 0.2% when α is changed to 0.65, Cohen’s kappa enhances by 3%, but accuracy of stage N1 decreases from 62 to 55%. We also divide the test data into two groups, namely stable epochs group and transitional epochs group. These groups present different optimal testing α. We infer that a minor α can make the model focus more on the SLB, thereby improving the performance of the model on transitive epochs, which contains fewer deterministic features. From a practical standpoint, if stage shifting is more concerned, set α < 0.5 during the testing phase. Conversely, if macroscopic sleep information is of greater interest, α can be set equal to or greater than 0.5. The subjects with varying degrees of obstructive sleep apnea have an insignificant effect on the model performance curves.

The cohorts used in this work have demographic diversity, consider a significant factor contributing to the statistical variability of sleep architectures, and are a challenge for model generalization. As discussed in (Olesen et al., 2020), more data is good and diverse data is better. This is confirmed by comparing the proposed model evaluation on SHHS and mixed-cohort. The mixed-cohort evaluation shows that the model performance does not deteriorate with the introduction of diverse data, but outperforms the result of the SHHS1 test. It is also demonstrated that the proposed model does not underfit due to the network structure limitations. As compared to other cohorts, MROS has significantly lower F1 scores for N3 which we considered that elder males have minimal N3 sleep, leading to high statistical fluctuations.

In clinical applications, machine learning models are generally required to process unseen data from different devices, subjects, and operating conditions. However, due to the homogenized model comparison framework and study datasets, cross-dataset validation has been overlooked by many methodological studies in the literatures, which is an effective means of assessing the generalization and robustness of a model by evaluating its performance on unseen datasets. We examined several studies that included cross-dataset validation. Zhang et al. (2019) achieved MF1 scores of 0.66 to 0.79 and Cohen’s Kappa of 0.53 to 0.70 in cross-dataset validation. Guillot and Thorey (2021) obtained direct transfer MF1 scores of 0.726 and 0.763 on MROS and SHHS, respectively. Xu et al. (2022) achieved the highest accuracy of 67.68% and Cohen’s Kappa of 0.4987 on unseen datasets. Even though a method developed on one cohort shows better performance as compared to human scoring agreement, its performance is significantly lower in cross-dataset/cohort tests (direct transfer), which typically requires fine-tuning with samples from the target cohorts. Encouragingly, in a recent similar study based on large-scale research subjects (Perslev et al., 2021), MF1 scores of 0.73 to 0.82 on eight hold-out datasets were obtained, with comparable direct transfer performance to ours. In our cross-dataset validation experiment, the proposed model achieves much higher MF1 scores on unseen cohorts as compared to other previous attempts discussed in literature and predicted higher levels of agreement as compared to human scores for each dataset.

The pathological differences also exist between subjects in a certain cohort. In the benchmark comparison for CFS, PSY group contains more light sleep (N1, N2), less deep sleep (N3), and almost the same REM sleep as compared to the HC group (Wake is not involved in statistics considering the operational differences of PSGs). More N1 sleep leads to an amplified problem of unbalanced prediction by the benchmark algorithm. When a biased predictor is applied on the PSG of psychiatric disorders, it leads to a more dysfunctional sleep structure for the whole night and affects the subsequent diagnosis. Contrary to the benchmark algorithm, the proposed algorithm achieves higher accuracy for the light sleep stages in PSY group, which is more applicable to the subjects with psychiatric disorders increasing light sleep and making the prediction results more informative.

This study also provides an idea of physiological signal processing oriented for difficult classes or low resource problems. Please note that abnormal or minority physiological features can be easily confused with other features, and are often difficult to obtain, such as the N1 sleep feature in sleep staging. The naive resampling causes the classifier to be trapped in its sensitivity and specificity balance. The transitive learning strategy proposed in this work gradually favors the low-resource features after fully trained universal features, using an end-to-end model.

The proposed work has several limitations. This study is conducted on publicly available cohorts derived from population-based studies and not disease-specific ones. The proposed algorithm is applicable to disease-related clinical data due to its more accurate N1 prediction. Although we perform a subgroup evaluation at CFS based on medical history, it still lacks a disease-specific dataset to support it. If there is a clinical sleep dataset with significant increase in N1 sleep, we can further validate the effectiveness of the proposed method on differentiated sleep structures. In this work, five specific PSG channels have been used as the input. It is expected that the model performance will be further improved, especially for scoring light sleep, by introducing frontal (spindle wave and K-complex predominate) and occipital (alpha wave predominate) regional EEGs.

In conclusion, this work provides a powerful tool for automatic sleep staging, which performs at the level of human scorers in each sleep/wake stage. It achieves more accurate stage N1 predictions with minimal impact on the overall performance. The pre-trained model is capable of directly performing sleep staging on unseen PSG and exhibits exceptional performance. The proposed method is conducive to expanding access to sleep-related diagnostics, especially those associated with increased N1 sleep.

## Data availability statement

Publicly available datasets were analyzed in this study. This data can be found at: https://sleepdata.org/datasets.

## Author contributions

DZ, YS, and YC: conceptualization, methodology, software, and writing original draft. JS: investigation and project administration. XZ: methodology and funding acquisition. LL, CT and NX: data curation, resources, and result interpretation. BC and WQ: supervision and funding acquisition. All authors contributed to the article and approved the submitted version.

## Funding

This work was funded by the Innovation Team and Talents Cultivation Program of National Administration of Traditional Chinese Medicine (Grand No. ZYYCXTD-C-202004), the National Key R&D Program of China (Grand No. 2021YFF0306500), theKey Research and Development Program of Shaanxi (Grand No. 2020ZDLSF04-05); Natural Science Basic Research Program of Shaanxi (Grand Nos. 2022JQ-649).

## Conflict of interest

The authors declare that the research was conducted in the absence of any commercial or financial relationships that could be construed as a potential conflict of interest.

## Publisher’s note

All claims expressed in this article are solely those of the authors and do not necessarily represent those of their affiliated organizations, or those of the publisher, the editors and the reviewers. Any product that may be evaluated in this article, or claim that may be made by its manufacturer, is not guaranteed or endorsed by the publisher.
