# Supplementary material for: A two-branch trade-off neural network for balanced scoring sleep stages on multiple cohorts
Source: Front Neurosci. 2023 Jun 23;17:1176551. doi: 10.3389/fnins.2023.1176551 (PMC10326279; doi:10.3389/fnins.2023.1176551)
Supplement: Supplementary file 1 [file Data_Sheet_1.docx]

Supplementary Material

A two-branch trade-off neural network for balanced scoring sleep stages on multiple cohorts

Di Zhang*^a,b^*, Jinbo Sun*^a,b,*^*, Yichong She*^a,b^*, Yapeng Cui*^a,b^*, Xiao Zeng*^a,b,*^*, Liming Lu*^c^*, Chunzhi Tang*^c^*, Nenggui Xu*^c^*, Badong Chen*^d^*,and Wei Qin*^a,b^*

^a^Engineering Research Center of Molecular and Neuro Imaging of the Ministry of Education, School of Life Science and Technology, Xidian University, Xi’an, Shaan xi 710126, China.

^b^Intelligent Non-invasive Neuromodulation Technology and Transformation Joint Laboratory, Xidian University, Xi'an, China.

^c^South China Research Center for Acupuncture and Moxibustion, Medical College of Acu-Moxi and Rehabilitation, Guangzhou University of Chinese Medicine, Guangzhou, China

^d^College of Artificial Intelligence, Xian Jiaotong University, Xian, Shaanxi, China

*** Correspondence:**

Corresponding Authors: Jinbo Sun and Xiao Zeng.

E-mail addresses: [sunjb@xidian.edu.cn](mailto:sunjb@xidian.edu.cn) (Sun J), xiaozeng1024@gmail.com (Zeng X)

# Supplementary Figures

**
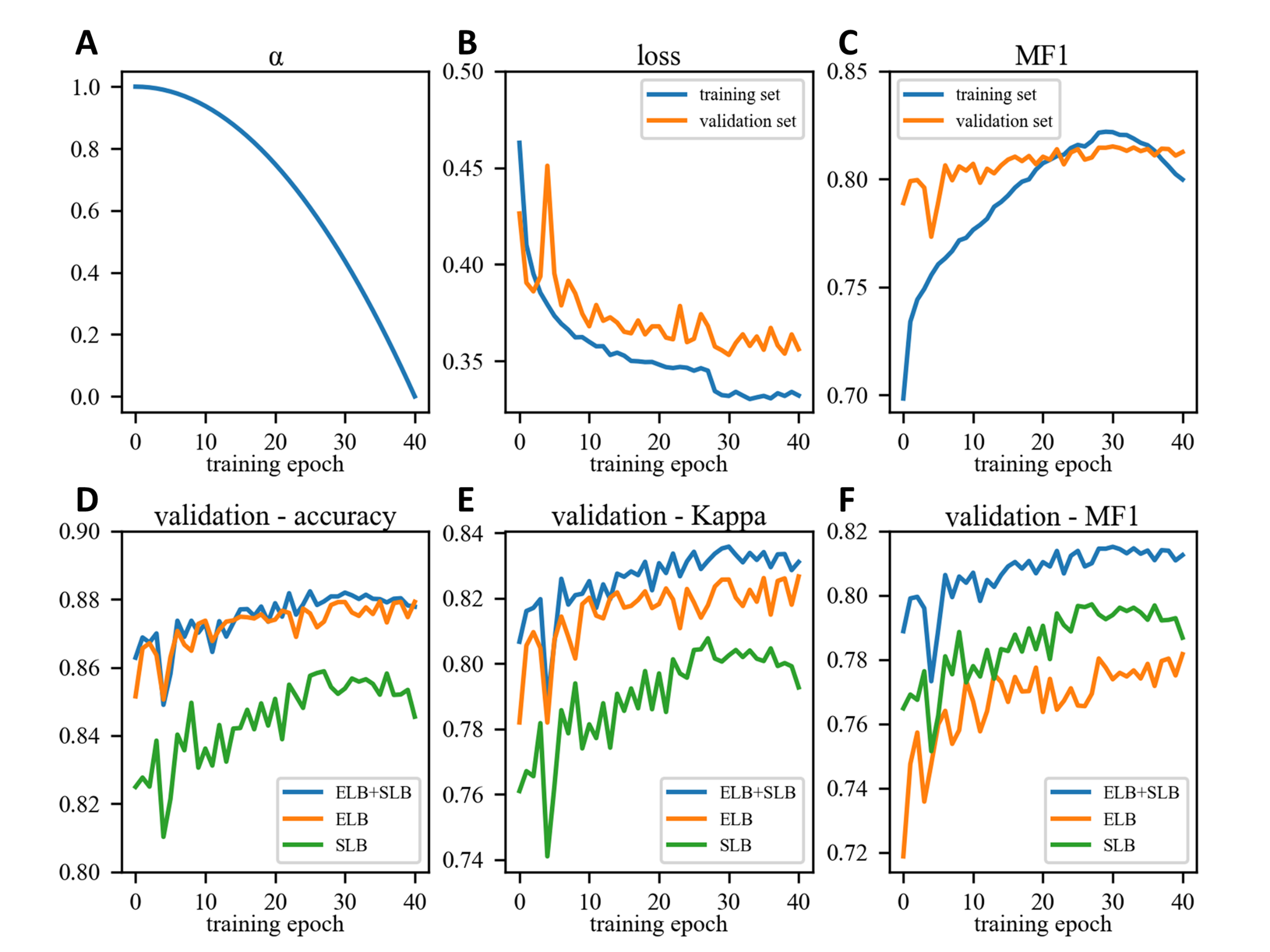
**

**FIGURE S1.** Curves of parameters or metrics as the process of model training. The horizontal axis represents the training epochs; each epoch contains 3500 mini-batch training iterations. (A) shows the α settings in proposed transitive learning strategy. (B, C) show the prediction loss values and MF1 scores on training and validation sets. (D, E, F) show the performance metrics on validation set when using the merged two-branch output and when using only single branch, where ELB represents epoch learning branch and SLB represents sequential learning branch.


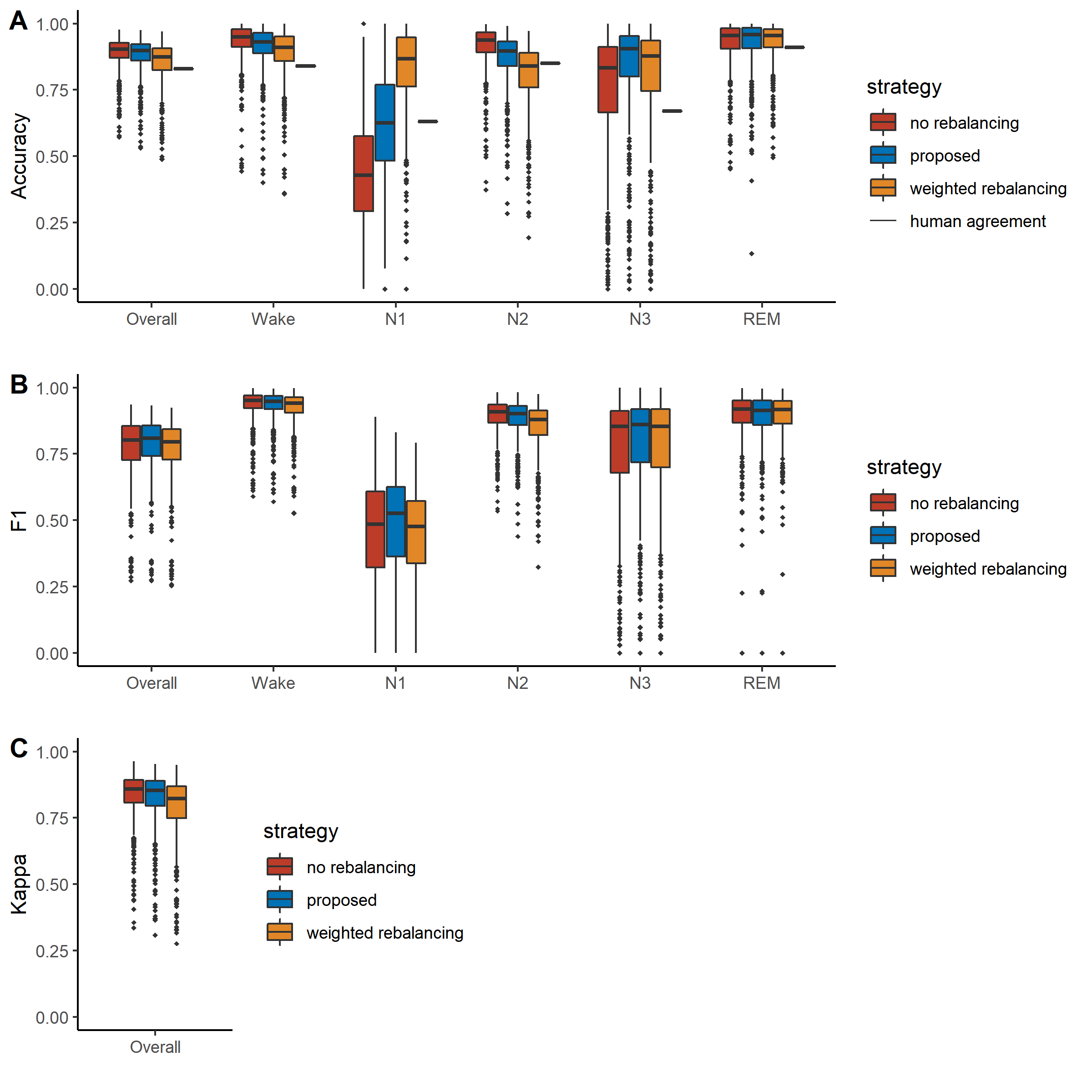


**FIGURE S2.** Boxplots illustrating the distributions of metrics for three models fitted with different rebalancing strategies: training with class imbalanced, training with proposed two-branch transitive training strategy, and training with class balanced. class imbalance represents training with a cross-entropy loss function whose weights are same, and class imbalanced represents training with a cross-entropy loss function whose weights are proportional to the inverse of the sample size for each class.


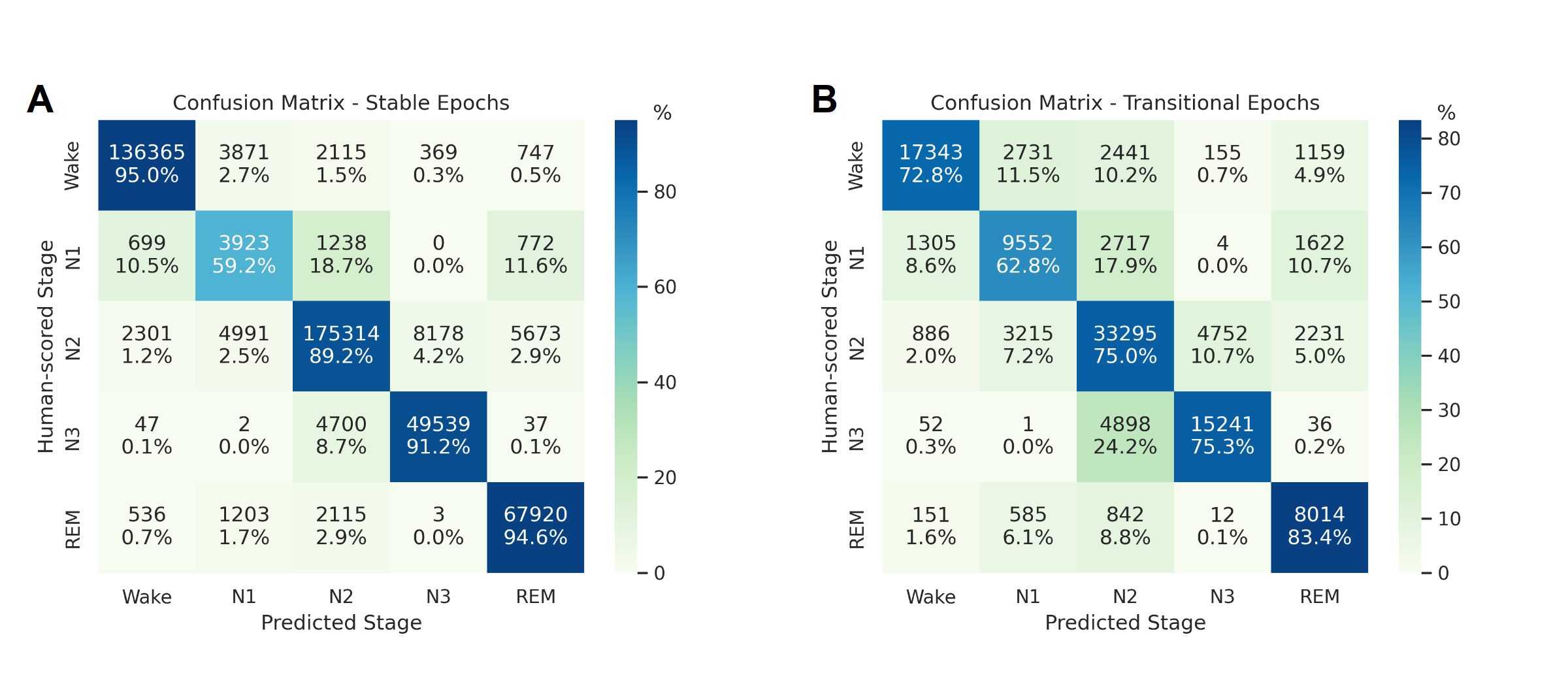


**FIGURE S3.** Confusion matrices for stable and transitional epochs. Model testing results are shown for two subsets of SHHS1 test set. Epochs were defined as stable epochs if their labels are the same as previous and subsequent epochs, otherwise they are defined as transitional epochs.


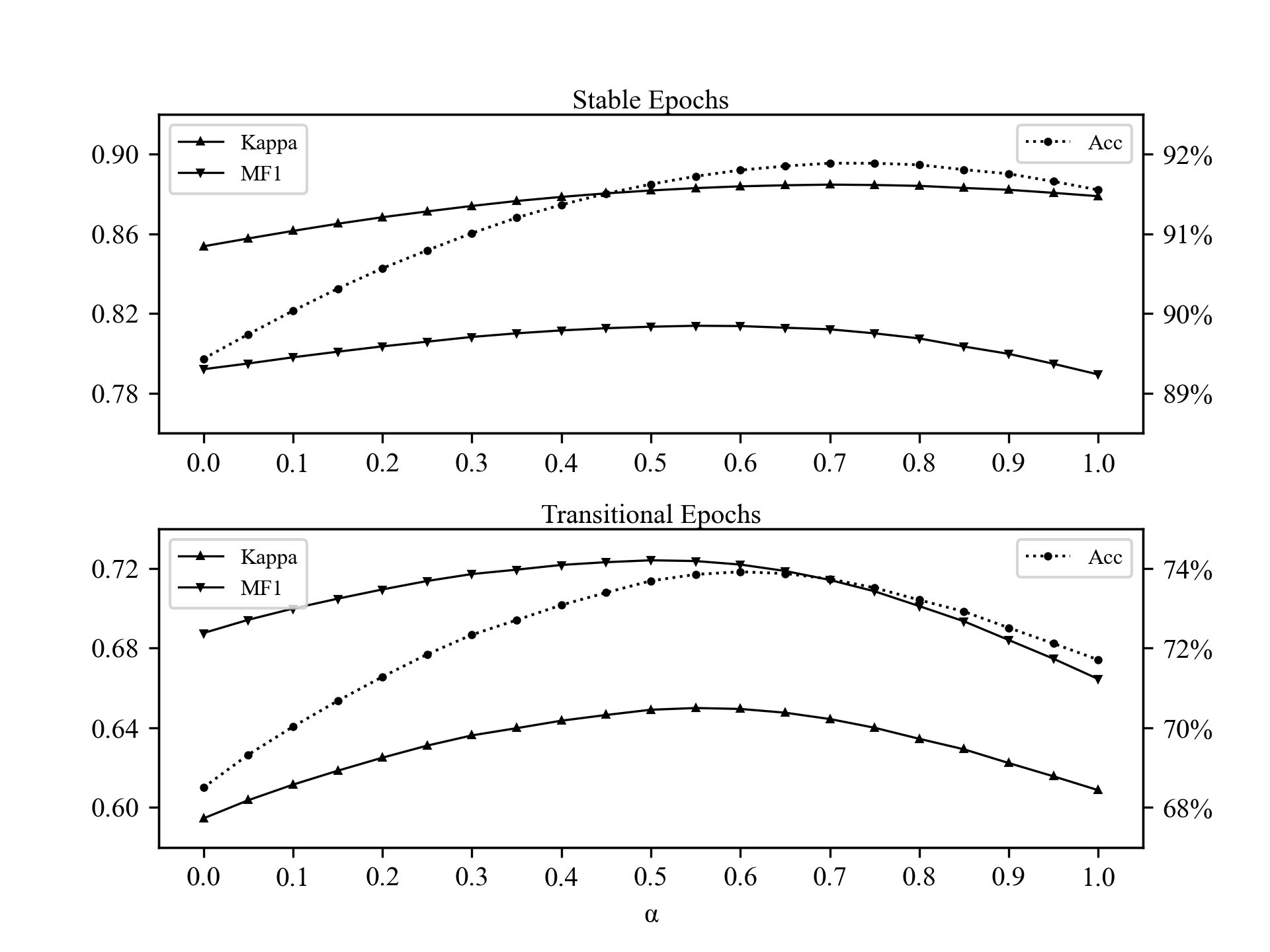


**FIGURE S4.** Model performance curves on stable and transitional epochs at different $\alpha$ settings. As the test $\alpha$ is set from zero to one, each model performance metric displays a first increase and then decrease pattern.


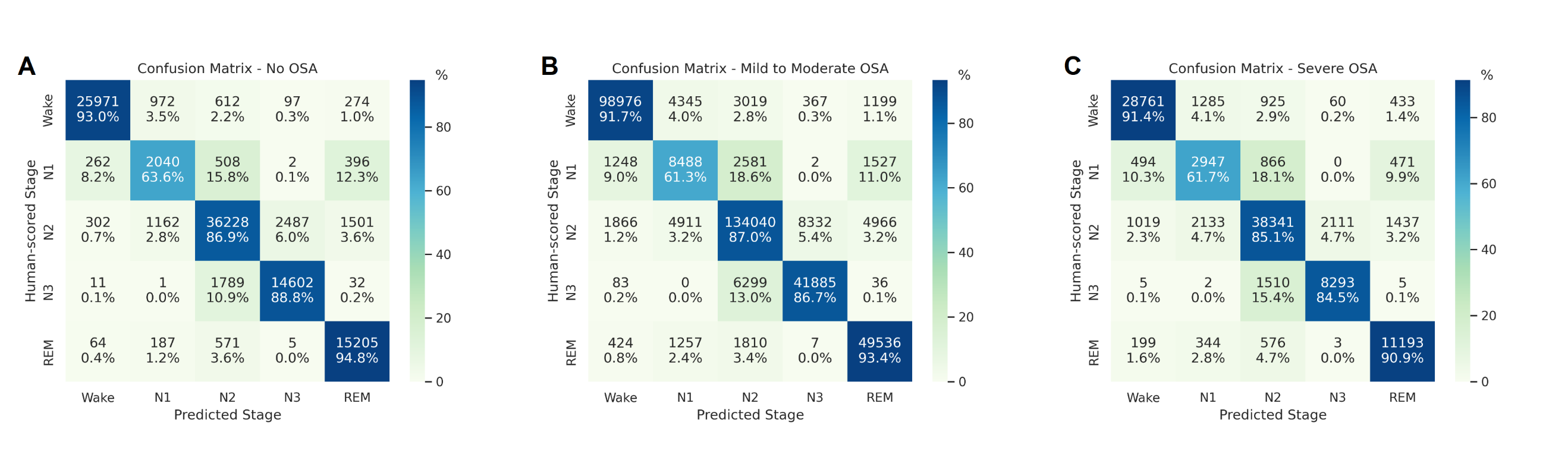


**FIGURE S5.** Confusion matrices for different obstructive sleep apnea severity subgroups. Model testing results are displayed for three subsets of SHHS1 test set, classified with varying degrees of obstructive sleep apnea (OSA), determined by each subject’s Apnea-Hypopnea Index (AHI). (No OSA: AHI<5, mild to moderate OSA:5≤AHI≤30, severe OSA: AHI≥30)


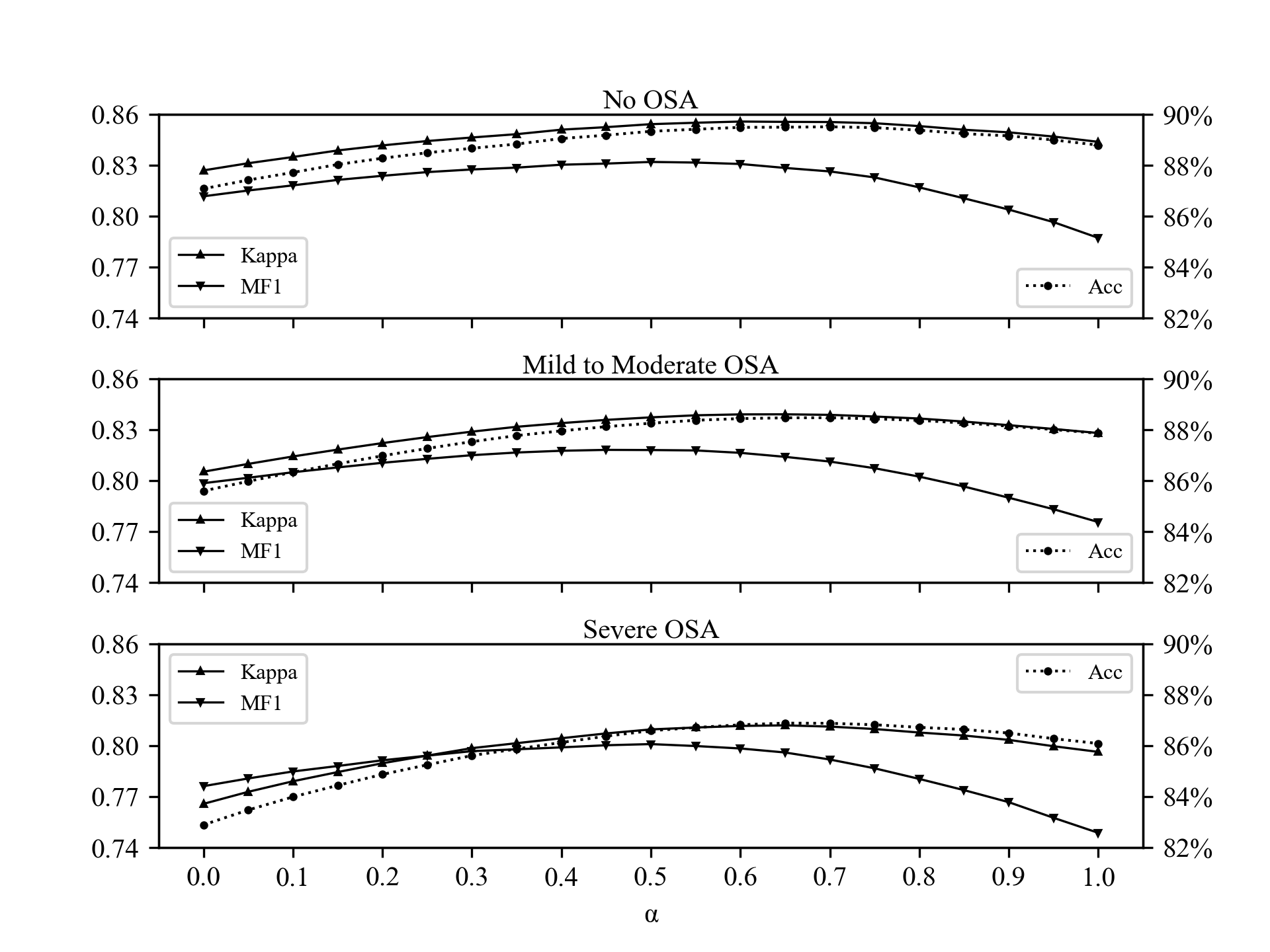


**FIGURE S6.** Model performance curves on three obstructive sleep apnea severity subgroups at different $\alpha$ settings. As the test $\alpha$ is set from zero to one, each model performance metric displays a first increase and then decrease pattern.


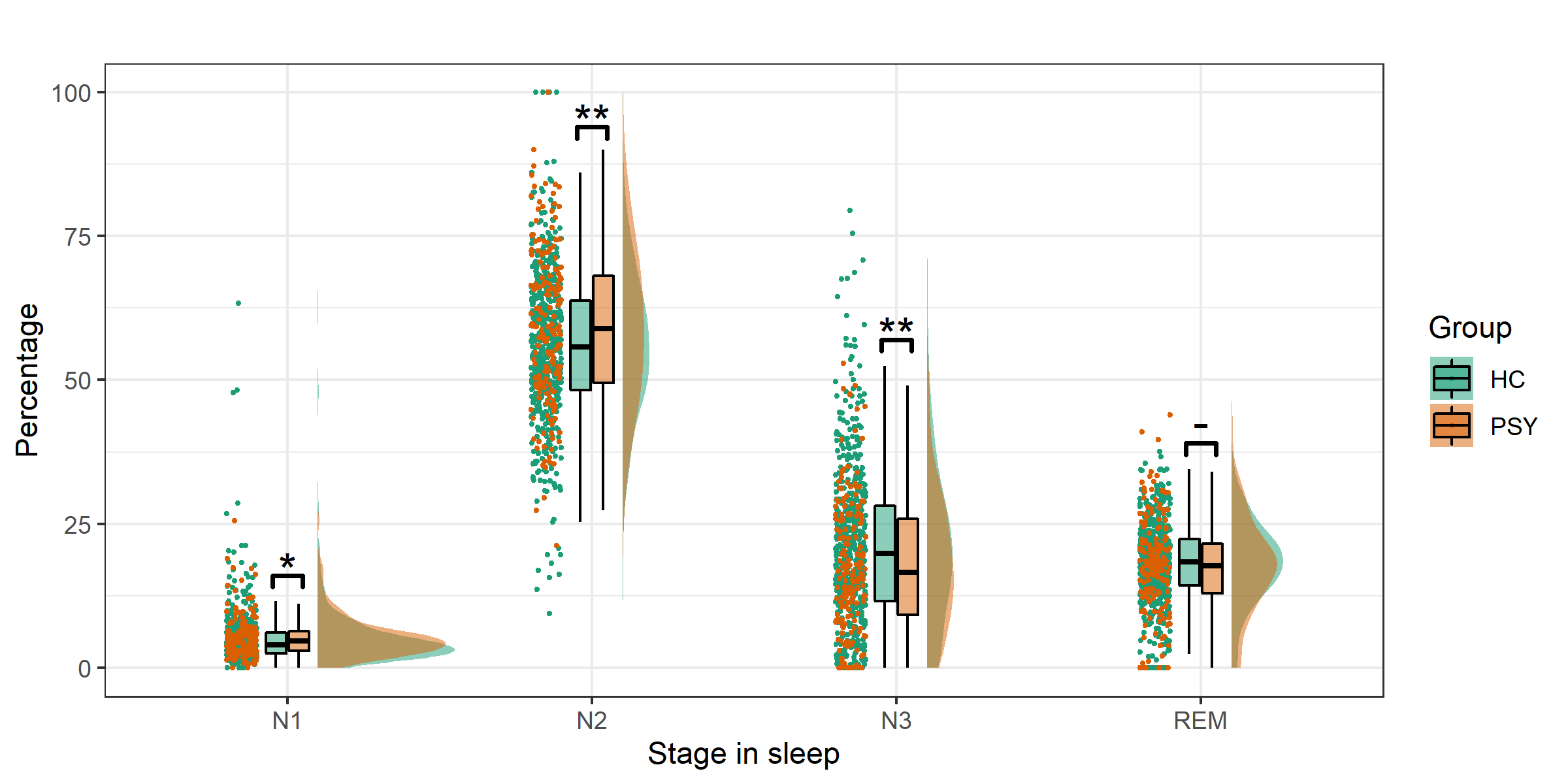


**FIGURE S7.** Percentage of each stage in total sleep for each subject in CFS. PSY: psychiatric disorders, HC: healthy controls, *: p<0.05, **: p<0.01, −: p>0.05.
